# Oxaliplatin-induced thrombotic microangiopathy: a case report

**DOI:** 10.1186/s13256-022-03309-7

**Published:** 2022-03-19

**Authors:** Rhea Saad, Audra Hannun, Sally Temraz, Antoine Finianos, Rony M. Zeenny

**Affiliations:** 1 American University of Beirut Medical Center, Beirut, Lebanon; 2 Medical University of South Carolina, Charleston, SC USA; 3 INSPECT-LB (Institut National de Santé Publique, d’Épidémiologie Clinique et de Toxicologie-Liban), Beirut, Lebanon

**Keywords:** Hemolytic uremic syndrome, Microangiopathic hemolytic anemia, Thrombotic microangiopathy, Oxaliplatin, Drug-induced thrombotic microangiopathy, Case report

## Abstract

**Background:**

Oxaliplatin-based chemotherapy represents a standard of care in the treatment of metastatic colorectal cancer. We report a rare case of fulminant oxaliplatin-induced thrombotic microangiopathy, clinically suggestive of hemolytic–uremic syndrome, occurring in a female patient with a prolonged history of exposure to oxaliplatin for the treatment of metastatic colon cancer.

**Case presentation:**

A 73-year-old Caucasian female with a treatment history including several lines of chemotherapy for the management of metastatic colon cancer was reinitiated on chemotherapy with oxaliplatin, fluorouracil, and leucovorin with bevacizumab for disease progression. She presented to the emergency department with malaise, headache, vomiting, and decreased urine output appearing a few hours after chemotherapy administration. Clinical symptoms and laboratory findings were suggestive of thrombotic microangiopathy, with a triad of microangiopathic hemolytic anemia, pronounced thrombocytopenia, and acute renal failure. The predominance of the severe renal failure was evocative of hemolytic–uremic syndrome. The rapid development of the thrombotic microangiopathy was linked to exposure to oxaliplatin. The patient was promptly managed with daily plasma exchange and high-dose corticosteroids, platelet, and red blood cell transfusions in conjunction with intermittent hemodialysis, and she recovered progressively.

**Conclusion:**

Our case confirms the risk of hemolytic–uremic syndrome as a rare and life-threatening complication of oxaliplatin-based chemotherapy. A dose-dependent, drug-induced toxicity mechanism is suggested. Physicians need to maintain a high level of clinical suspicion to diagnose and treat this acute life-threatening disorder.

## Background

Thrombotic microangiopathies (TMAs) are a group of acute microvascular occlusive disorders recognized by the triad microangiopathic hemolytic anemia (MAHA), thrombocytopenia, and microvascular thrombosis with typical vessel wall abnormalities [1, 2]. Thrombotic thrombocytopenic purpura (TTP) is a MAHA with moderate to severe thrombocytopenia, where the systemic formation of platelet aggregates causes ischemia in the brain and other organs. It is characterized by organ dysfunction, including neurological changes and abnormalities in the heart, pancreas, thyroid, adrenal glands, intestinal mucosa, and other tissues. Kidney function abnormalities are minimal [3]. The diagnosis of TTP is confirmed by a severe deficiency of ADAMTS-13 activity (< 10%) [3, 4]. Hemolytic–uremic syndrome (HUS) is a TMA that usually affects the kidneys, with platelet–fibrin thrombi occluding predominantly the renal circulatio﻿n [5]. Renal failure dominating the clinical picture is highly suggestive of HUS [3]. Despite the overlapping clinical manifestations of both disorders, a diagnosis must be performed to guide treatment decisions. Differential diagnosis should also be performed with disseminated intravascular coagulation (DIC) [6]. TMA is a medical emergency where understanding the potential etiology can be critical for diagnosis and appropriate management [2].

Several drugs and other substances such as vaccines, herbal remedies, toxins, and illegal drugs have been associated with TMA [7]. The syndrome is described as drug-induced TMA (DITMA) [7]. Two mechanisms are suggested for DITMA [8, 9]. The first one is an idiosyncratic, acute, immune-mediated etiology due to drug-dependent antibodies that attack platelets, neutrophils, endothelial cells, and other cells [7–9]. Quinine represents the drug most commonly associated with this type of TMA, with documentation of quinine-dependent antibodies [8]. The second mechanism is a direct toxic effect due to direct cellular damage [8, 9], which might be acute, dose-dependent toxicity or chronic toxicity related to the cumulative dose and duration of the drug [8, 9]. Four classes of drugs have been associated with dose-dependent, toxicity-mediated TMA: chemotherapy agents (for example, gemcitabine, mitomycin C), immunosuppressive agents (for example, cyclosporine, tacrolimus), vascular endothelial growth factor inhibitors (for example, bevacizumab), and opioids (for example, oxymorphone) [7, 10].

Oxaliplatin is a drug commonly used in the treatment of advanced colon cancer. Few cases of HUS associated with the use of oxaliplatin have been reported in the literature [11–13]. This report describes a rare life-threatening case of oxaliplatin-induced TMA, with a clinical and laboratory presentation suggestive of HUS, in a patient with metastatic colon cancer.

## Case presentation

A 73-year-old Caucasian female, never smoker, with a past medical history of hypertension was diagnosed with stage IV colon adenocarcinoma with liver metastasis in October 2008. She received chemotherapy with capecitabine, leucovorin, and oxaliplatin (XELOX) and bevacizumab for five cycles, then underwent a right hemicolectomy and a partial hepatectomy followed by maintenance therapy with bevacizumab. In February 2011, she was initiated on fluorouracil, leucovorin, and irinotecan (FOLFIRI) and cetuximab for disease progression in the liver. In May 2011, she underwent a resection of the new hepatic lesion followed by intra-arterial chemoembolization sessions for 3 months. Three years later, in August 2014, a positron emission tomography–computed tomography (PET–CT) scan showed evidence of metastatic retroperitoneal lymphadenopathy, consistent with disease recurrence. She was then initiated on mFOLFOX-6 with bevacizumab with a good imaging response, followed by a year of maintenance therapy with fluorouracil, leucovorin, and bevacizumab. In December 2016, her treatment plan was switched to fluorouracil, leucovorin, and irinotecan (FOLFIRI) for disease progression to the lungs, followed by maintenance therapy with fluorouracil, leucovorin, and bevacizumab. A few months later, in August 2017, a PET–CT scan showed a new lung nodule. She was then shifted to mFOLFOX6 with panitumumab followed by maintenance therapy with fluorouracil and leucovorin. In May 2018, the treatment protocol was changed to FOLFIRI with cetuximab, until a new disease progression in the lymph nodes, and then around the hepatic artery stent, occurred in January 2019. The patient was then reinitiated on mFOLFOX6 with bevacizumab. She received her first cycle on 21 February and later experienced an episode of neutropenia for which the second cycle was delayed. On 11 March, she received her second cycle of chemotherapy with a 25% dose reduction.

On 13 March, she presented to the emergency department with fatigue, malaise, orbital headache, nausea and vomiting, mild abdominal pain, and chills. She stated that her symptoms started hours after the chemotherapy infusion and reported a decrease in urine output and a darkening of urine. On physical examination, the patient had jaundice with icteric sclera since a day ago. Her vitals showed a temperature of 37.1 °C, a heart rate of 88 beats per minute, a respiratory rate of 18 breaths per minute, a blood pressure of 16.7/7.2 mmHg, and oxygen saturation of 100%. A cardiac workup ruled out acute myocardial infarction. The initial blood workup was significant for thrombocytopenia (platelet count 30.10^3^ cells/mm^3^) and anemia (hemoglobin 8.1 mg/dL). The anemia was defined as hemolytic by markedly increased bilirubin (total bilirubin 6.4 mg/dL, indirect bilirubin 3.3 mg/dL), high aspartate aminotransferase (AST) (1725 IU/L), high lactate dehydrogenase (LDH) (4866 IU/L), and low haptoglobin (< 0.1 g/L). The patient also showed signs of acute renal failure (serum creatinine had markedly increased from 0.9 to 5.5 mg/dL within a week, blood urea nitrogen (BUN) 78 mg/dL, and uric acid 10.6 mg/dL). The blood film inspection showed signs of hemolysis with a slight anisopoikilocytosis, slight hypochromia, some ovalocytes and echinocytes, few schistocytes and helmet cells, rare teardrop red blood cells, and rare stomatocytes. There were also occasional polychromatophilic red blood cells (reticulocytosis). Antibody screening was negative, confirming the MAHA diagnosis. The coagulation panel was normal. Relevant laboratory parameters are documented in Table 1. Her chronic medications included irbesartan/amlodipine and atenolol.

**Table 1:** Summary of relevant laboratory parameters

Timepoint	Pre-chemotherapy	Day 1 (ED)	Day 2	Day 3	Day 4	Day 5	Day 6	Day 7	Day 8	Day 9	Day 10	Day 11	Day 12	Day 13	Day 14	Day 15	Day 22	Day 27
Adjusted WBCs (per mm^3^)	11,000	9800	6337	3900	2574	4600	6400	7000	7100	7900	6600	6400	6400	5600	4800	4700	4900	7000
Hemoglobin (g/dL)	11.1	8.9	8.1	7.9	8.1	7.5	8.6	7.8	8.4	7.8	6.7	9.5	10.4	9.8	9.8	9.5	8	9.1
Platelets (×10^3^/mm^3^)	301	33.8	30	10	7.5	13.1	39.4	78.8	91.9	61	69	80	104	117	121	134	121	116
Creatinine (mg/dL)	0.9	5.5	5.8	4.9	3.7	3.1	3	2.9	4.3	3.1	3.1	3.1	3.9	3.9	3.2	2.6	1.2	1.1
Total/direct bilirubin (mg/dL)	0.4/0.1	3.3/2.2	1.8/1	1.4/0.6	1.1/0.4		0.8/0.3				0.6/0.2			0.7/0.3		0.5/0.2		
AST (IU/L)	26	1725	347	83	38		38											
ALT (IU/L)	19	930	294	99	49		39											
Haptoglobin (g/L)	< 0.1	0.52	0.22	0.49	0.59	0.55	0.68	0.6	0.54	0.69	0.82			0.94		0.94		1.31
LDH (IU/L)	237	4866	1093	429	265	283	297	266	266	249	224	219	231	221		161	148	159

*ED* emergency department, *WBCs* white blood cells, *AST* aspartate aminotransferase, *ALT* alanine aminotransferase, *LDH* lactate dehydrogenase, *IU* international unit

The clinical laboratory findings were consistent with thrombotic microangiopathy (TMA). An ADAMTS13 activity test was performed to investigate a potential TTP, whereas the significant picture of the predominance of renal failure was more suggestive of HUS. The occurrence of TMA after the chemotherapy session was suggestive of drug-induced thrombotic microangiopathy (DITMA), notably related to the administration of a chemotherapy agent.

Treatment was started promptly with daily plasma exchange and immunosuppression with methylprednisolone (1 mg/kg/day) until a diagnosis of TTP was ruled out, then tapered later on. She also received packed red blood cell transfusions and platelet transfusions, and underwent daily intermittent hemodialysis. Electrolyte imbalances were also managed. Seven days later, the patient’s laboratory studies (Table 1) showed a stable hemoglobin level, a much-improved platelet count, and no clinical evidence of hemolysis. Her platelet count normalized on day 12. Daily plasma exchange was continued for eight consecutive days, and dialysis for eight consecutive days. The patient was transferred to another medical center in the Kingdom of Saudi Arabia (KSA) for continuity of care and possible treatment of the DITMA with eculizumab.

## Discussion

Oxaliplatin, a third-generation platinum derivative, is a commonly used agent in the treatment of metastatic colorectal cancer, notably in combination with fluorouracil and leucovorin (FOLFOX protocol) as well as with antiangiogenic targeted therapies [14]. Oxaliplatin has been associated with few cases of acute thrombocytopenia and TMA published in the literature, including three cases of HUS and three cases of TTP [11–13, 15–18]. Dahabreh *et al*. [11] reported in 2006 a case of HUS following the fourth cycle of oxaliplatin-based adjuvant chemotherapy in a 52-year-old man. Phan *et al*. [12] described in 2009 a case of oxaliplatin-induced acute renal failure due to tubular necrosis, clinically presenting as TMA, in a 65-year-old man. In 2011, Racca *et al*. [13] reported a case of HUS in a 49-year-old female associated with prolonged oxaliplatin exposure in a patient with colon cancer. As for TTP, Niu and Mims [16] and Lucchesi *et al*. [17] described in 2012 and 2013 cases of oxaliplatin-induced TTP in two 68-year-old women receiving chemotherapy for progression of metastatic colon cancer. Baretta *et al*. [18] in 2013 reported a case of fatal TTP in a 77-year-old man treated with FOLFOX chemotherapy for progression of advanced colon cancer.

This report presents a case of oxaliplatin-induced TMA, clinically suggestive of HUS, occurring in a 73-year-old female with a long history of exposure to oxaliplatin in her multiple-line treatment history for advanced colon cancer. The patient was reinitiated on the mFOLFOX6 regimen for the fourth time (2008, 2014, 2017, and 2019) and was on the second cycle of her current course.

Our patient presented with the classical triad of MAHA, thrombocytopenia, and renal failure, consistent with the diagnosis of TMA. The picture of a predominant renal failure was suggestive of HUS. The negative antibody screening and normal coagulation panel also confirmed the diagnosis. The normal ADAMTS-13 activity revealed several days after the patient’s admission made a diagnosis of TTP unlikely. Neurological changes suggestive of TTP were also absent. Patients with DITMA often present with a sudden onset of systemic symptoms, with anuric acute kidney injury, manifesting hours after drug exposure. Symptoms commonly include chills, fever, abdominal pain, diarrhea, nausea, and vomiting. Our patient had a typical presentation of DITMA occurring after exposure to oxaliplatin with systemic symptoms and severe acute kidney injury. On Naranjo’s causality assessment scale, the adverse event was 6, indicating a “probable” reaction to oxaliplatin (Table 2). Testing for antibodies against the complement fraction C3 to confirm the diagnosis of HUS, as well as testing for oxaliplatin-dependent antibodies to confirm a definite association with TMA was not possible owing to unavailability of the assays.

**Table 2:** Naranjo algorithm—adverse drug reaction probability scale

Question	Yes	No	Do not know	Score
1. Are there previous conclusive reports on this reaction? [12–18]	+1	0	0	+1
2. Did the adverse event appear after the suspected drug was administered?	+2	−1	0	+2
3. Did the adverse event improve when the drug was discontinued or a specific antagonist was administered?	+1	0	0	+1
4. Did the adverse event reappear when the drug was readministered?	+2	−1	0	0
5. Are there alternative causes that could on their own have caused the reaction?	−1	+2	0	+2
6. Did the reaction reappear when a placebo was given?	−1	+1	0	0
7. Was the drug detected in blood or other fluids in concentrations known to be toxic?	+1	0	0	0
8. Was the reaction more severe when the dose was increased or less severe when the dose was decreased?	+1	0	0	0
9. Did the patient have a similar reaction to the same or similar drugs in any previous exposure?	+1	0	0	0
10. Was the adverse event confirmed by any objective evidence?	+1	0	0	0
	Total score: 6			

Another diagnostic challenge was the delay in receiving the result of the ADAMTS-13 activity due to the unavailability of the test in the country. The negative result was obtained after 14 days, during which plasmapheresis was initiated as a preemptive treatment for TTP. The American Society for Apheresis (ASFA) mentions the absence of a clear rationale for a benefit of plasmapheresis in DITMA, notably with the availability and efficacy data of eculizumab in this setting [19]. The guideline provides an evidence-based categorization of the benefit of plasmapheresis, with drugs such as quinine and gemcitabine being considered category IV (plasmapheresis ineffective or harmful) [19]. However, it states that plasmapheresis may be appropriate when there is uncertainty about the diagnosis of DITMA versus TTP [19], which was the case for our patient.

Oxaliplatin was presumed the causative element rather than bevacizumab since our case shared elements more consistent with the reports of oxaliplatin-induced TMA found in the literature [11–13, 15–18]. The first distinguishing point was the clinical presentation with a rapid onset of symptoms, severe thrombocytopenia, severe hemolytic anemia, and renal failure occurring hours after chemotherapy administration. This seemed more characteristic of a typical chemotherapy-related TMA, rather than a monoclonal antibody-related TMA. In case reports of oxaliplatin-induced TMAs, notably HUS [11, 12], the patients had acute life-threatening symptoms appearing hours after oxaliplatin administration, with pronounced thrombocytopenia, severe hemolytic anemia, and renal failure. On the other hand, although limited cases are published, three case reports of bevacizumab-induced atypical HUS were described by Vakiti *et al*. [20] These cases share similar features of a TMA developing progressively, over several weeks to months after the administration of bevacizumab, with mild acute renal failure, no life-threatening hemolytic anemia, and platelet level always above 50,000. Eremina *et al*. [21] described six cases of TMA resembling HUS following bevacizumab administration. However, beyond the consistent (sometimes mild) renal injury, probably due to vascular endothelial growth factor (VEGF) inhibition, the clinical presentation was not uniform across all patients: while some had anemia without schistocytes, others had no anemia reported at all. There were no severe life-threatening acute symptoms after chemotherapy administration, but rather a renal injury developing over weeks.

Another characteristic is the previous exposure of the patient to cumulative doses of oxaliplatin, which corroborates the literature finding that oxaliplatin-induced HUS occurred in patients receiving more administrations of the drug than the foreknown standard schedule. It often appeared in patients with a long history of exposure to oxaliplatin after receiving several courses over the lifespan [11, 12].

Antibody-mediated hemolysis and/or thrombocytopenia associated with oxaliplatin-based chemotherapy is a possible etiology for clinical presentations similar to the one of our patient [7]. However, such pathogenesis [7] would be associated with a positive direct agglutination test, which was not the case in our report. Such a presentation is most likely in a case of dose-dependent, toxicity-mediated DITMA, where medications such as chemotherapy agents, immunosuppressive agents, and opioids can cause DITMA syndromes due to direct cellular damage [9]. This could explain the rapid onset of symptoms, rather than bevacizumab-induced TMA, which develops due to a disruption of the VEGF function [7].

TMAs can be potentially fatal in the absence of prompt treatment and supportive care. Clinical response is defined as sustained normalization of platelet counts above the lower limit of the established reference range (for example, > 150.10^3^/mm^3^ for two consecutive days) and of LDH after cessation of plasma exchange [22, 23]. Eculizumab, a complement C5 inhibitor, is to date the only approved treatment for patients with atypical HUS [24]. Due to the unavailability of the drug, the patient was transferred to a medical center in KSA for continuity of care.

## Conclusion

In conclusion, we report a case of oxaliplatin-induced TMA, with a clinical and laboratory presentation suggestive of HUS, in a patient with metastatic colon cancer. The report confirms the association between oxaliplatin and TMA, a rare side effect that was mentioned in few other literature publications. We suggest that the etiology of this case of TMA was a dose-dependent, toxicity-mediated, drug-induced TMA due to direct cellular damage, although immune memory, tumor histology, and other unknown factors could also be responsible for HUS, which development must be taken into account in the decision-making process. Physicians need to maintain a high level of suspicion, notably in patients with a long history of exposure to oxaliplatin, to diagnose and treat this acute life-threatening disorder promptly.

## Data Availability

All data generated or analyzed during this study are included in this published article.

## Abbreviations

TMA: Thrombotic microangiopathy
MAHA: Microangiopathic hemolytic anemia
TTP: Thrombotic thrombocytopenic purpura
HUS: Hemolytic–uremic syndrome
DIC: Disseminated intravascular coagulation
DITMA: Drug-induced thrombotic microangiopathy
PET: Positron emission tomography
CT: Computed tomography
AST: Aspartate aminotransferase
LDH: Lactate dehydrogenase
